# Knock-down and speed of kill of a combination of fipronil and permethrin for the prevention of *Ctenocephalides felis* flea infestation in dogs

**DOI:** 10.1186/s13071-016-1345-4

**Published:** 2016-02-02

**Authors:** Lénaïg Halos, Josephus J. Fourie, Becky Fankhauser, Frederic Beugnet

**Affiliations:** Merial S.A.S., 29 Av Tony Garnier, 69007 Lyon, France; ClinVet International (Pty) Ltd, P.O. Box 11186, Universitas, Bloemfontein, 9321 Republic of South Africa; Merial Limited, 3239 Satellite Blvd, Duluth, GA 30096 USA

**Keywords:** Dogs, Fleas, *Ctenocephalides felis*, Fipronil, Permethrin, Speed of kill, Knock-down effect

## Abstract

**Background:**

A topical combination of fipronil + permethrin (Frontline Tri-Act®/Frontect®, Merial) has recently been developed to control fleas, ticks, mosquitoes, sandflies and stable flies on dogs. Two studies were conducted to assess its speed of kill and knock-down effect on *Ctenocephalides felis* fleas. The combination was compared to either fipronil alone or to a combination of permethrin, dinotefuran, and pyriproxyfen,

**Methods:**

In each study, 18 dogs were randomly allocated to one of three groups: (Group 1: untreated dog; Group 2: treated once on D0 with the combination of fipronil and permethrin; Group 3: treated once on D0 either with fipronil alone (study 1) or with a combination of permethrin, dinetofuran and pyriproxyfen (study 2)). Each dog was infested with 100 unfed adult *C. felis* fleas on Days 2 (study 2), 7, 14, 21 and 28. Fleas were collected from dogs at 1 h and 12 h post- infestations (PI) (study 1) or at 2 h and 6 h PI (study 2) to assess efficacy and from collection pans underneath cages 1 h (study 1) or 5 min (study 2) PI to assess knock-down effect.

**Results:**

All treated dogs had significantly (*p* ≤ 0.01) lower flea counts than untreated dogs at every time point in both studies. For a whole month, a significant knock-down effect against infesting fleas is obtained in five minutes PI with the combination of permethrin and fipronil. Complete efficacy (>95 %) was achieved in 1 h (study 1) or 2 h (study 2) PI for 14 days and by 6 h PI for all challenges conducted throughout the month. Efficacy remains >85 % at 2 h PI for the whole month. A significantly higher efficacy of the fipronil + permethrin combination compared to other treatments was demonstrated at the earliest time points for the month (1 h knock-down effect and insecticidal efficacy compared to fipronil alone; 5 min knock-down effect compared to the combination of permethrin + dinetofuran + pyriproxyfen).

**Conclusions:**

The rapid flea knock-down effect and speed of kill demonstrated by the spot on combination of fipronil + permethrin provide a reliable strategy against flea infestations in dogs.

## Background

Besides being the most common ectoparasites of pets worldwide [1], *Ctenocephalides* sp. flea burdens in dogs may lead to the development of hypersensitivity to flea bites and related allergic dermatitis (FAD) resulting in one of the most common forms of allergic of dermatitis in dogs [2, 3] and the risk for transmission of vector-borne pathogens such as *Rickettsia* spp., *Bartonella* sp. [4–6]. A desirable attribute of a highly effective flea control product is not only to treat and remove an existing infestation but more importantly, to protect the animal from re-infestation and potential concomitant vector-borne disease transmission or development of FAD. The key characteristics that a product should exhibit to achieve this objective include a rapid onset of action (speed of kill) and a sustained efficacy between administrations in order to limit blood intake. It has been demonstrated that fleas start to feed as early as 5 min post infestation [7–9]. It is therefore useful to evaluate the efficacy of products at early time points, soon after fleas have infested the animal.

A spot-on formulation of fipronil and permethrin (Frontline Tri-Act®/Frontect®) has recently been developed for use as a monthly topical solution for the control of fleas, ticks, mosquitoes, sandflies and biting flies in dogs. Initial efficacy studies demonstrated pulicidal activity for a full month [10], although the evaluations in these studies were based on 24 h counts following classic efficacy guidelines required to support registration of flea control products [11, 12].

According to the known properties of fipronil and permethrin, it was suspected that a combination of both molecules could provide increased insecticidal efficacy especially at early time points. Fipronil belongs to the phenylpyrazole family. It acts at synaptic ligand-gated chloride channels [13], where its action blocks transfer of chloride ions across cell membranes and results in uncontrolled activity of the central nervous system and death of parasites. Permethrin belongs to the Type I class of pyrethroids, which are acaricides and insecticides with repellent activity. Permethrin acts on voltage-gated sodium channels in the nerve cell membrane. It induces nerve cell membrane depolarization resulting in a sudden shock, known as the ‘knock-down’ effect. Affected arthropods stop all movement and appear dead although this knock-down can be reversible [13].

Permethrin alone is known for its short-lasting knock-down effect against *Ctenocephalides felis* fleas on dogs but its insecticidal efficacy doesn’t always attain the levels of efficacy required to support registration. This was confirmed during the development of the product where the efficacy of permethrin alone against *C. felis* was demonstrated to be between 23.7 and 86 % for the whole month (24 h counts), while the combination of permethrin/fipronil provided 98.4 to 100 % efficacy for the full month (*data not shown, Frontline Tri-Act® registration dossier*). A limited additive effect on the sustained pulicidal efficacy of the two molecules was thus expected. Further studies against flea infestations conducted with the combination product since its registration have demonstrated an improved sustained efficacy, especially in terms of speed of action. For example, Complete (>95 %) *Ctenocephalides canis* flea preventive efficacy was demonstrated to be >98.3 % as soon as 6 h post infestation (PI) for a whole month while a complete efficacy (>95 %) in 1 h PI was demonstrated for a week [14].

These results indicate that the 2 molecules may act synergistically. Synergy is defined as the interaction of two substances to produce a combined effect greater than the sum of their separate effects. For fipronil and permethrin, this implies that the flea activity of their combination should be greater than the efficacy of each active if they were given separately. The objective of the present studies were to demonstrate the speed of kill and knock-down effect on *C. felis* fleas of the combination of fipronil and permethrin in Frontline TriAct®/Frontect® when compared either to fipronil alone or to another insecticidal permethrin-based combination and for which a synergistic effect is described with the other insecticidal component, dinetofuran [15].

## Methods

### Animals and study design

Animals were healthy, pure or mixed-breed dogs of both sexes. Two studies were conducted under a controlled and blinded design, with dogs randomly allocated to three groups of six and eight dogs each in studies 1 and 2, respectively (control, treated with fipronil/permethrin combination, treated with Reference Product (either fipronil alone (study 1) or the combination of dinetofuran + permethrin + pyriproxyfen (study 2)). Prior to treatment, all dogs underwent a physical examination conducted by a veterinarian to ensure that they were healthy. To detect any treatment-related or unrelated adverse event, health observations were conducted daily from the start to end of all studies as well as at hourly intervals for 4 h immediately after treatment. All animals were managed similarly; with due regard for their well-being and in compliance with the Merial Ethics Committee and Merial Institutional Animal Care and Use Committee requirements.

The studies were designed in accordance with the *World Association for the Advancement of Veterinary Parasitology (WAAVP) guidelines for evaluating the efficacy of parasiticides for the treatment, prevention and control of flea and tick infestation on dogs and cats* [12], the EMEA guidelines [11] and were conducted in accordance with Good Clinical Practices as described in *International Cooperation on Harmonisation of Technical Requirements for Registration* of *Veterinary Medicinal Products* [16]. Both studies were conducted in South Africa.

### Flea strains

A laboratory bred strain (European strain) of *Ctenocephalides felis* (routinely fed on cats) was used for all infestations in both studies.

### Treatment

1. Dogs assigned to the control group 1 were not treated. On study Day 0, each dog in the treated group 2 received a topical application of the combination of permethrin/fipronil (Frontect®^M^/Frontline Tri-Act®) at the commercial dose of the product based on bodyweight (pipette dose) as per label instruction. Each dog in the treated group 3 received a topical application of fipronil (Effipro®, Virbac, Carros) at the commercial dose of the product based on bodyweight (pipette dose) as per label instruction.
2. Dogs assigned to the control group 1 were not treated. On study Day 0, each dog in the treated group 2 received a topical application of the combination of permethrin/fipronil (Frontect®/Frontline Tri-Act®) at the commercial dose of the product based on bodyweight (pipette dose) as per label instruction. Each dog in the treated group 3 received a topical application of dinetofuran + permethrin + pyriproxyfen (Vectra 3D®, Ceva, Libourne) at the commercial dose of the product based on bodyweight (pipette dose) as per label instruction.

#### Flea infestations, flea counts on dogs and underneath cages

1. Each dog was infested in an individual cage with 100 (±5) unfed adult fleas on Days 7, 14, 21 and 28. The dogs were comb-examined to collect and count live, moribund and dead fleas at 1 h and 12 h post- infestations (PI). At 1 h, the live fleas were placed back on their dog. Fleas were removed after the 12 h count. Fleas were also collected from a collection pan underneath cages 1 h after infestation and evaluated for viability.
2. Each dog was infested in an individual cage with 100 (±5) unfed adult fleas on Days 2, 7, 14, 21 and 28. The dogs were comb-examined to collect and count live, moribund and dead fleas at 2 h and 6 h PI. At 2 h, the live fleas were placed back on their dog. Fleas were removed after the 6 h count. Fleas were also collected on a plate put under the cage for 5 min after flea infestation in order to evaluate the knock-down effect (i.e. dead and moribund at 5 min).

In both studies, fleas were identified as dead if they were immobile. Fleas were defined as moribund if they were dying, i.e., trembling, convulsing, and not able to move normally. Moribund fleas were added to the dead fleas for the evaluation of the knock-down effect, whereas the number of live fleas on dogs was used to calculate efficacy, following the WAAVP and registration agencies guidelines.

### Data analysis

#### Speed of kill

In each study, efficacy against fleas was calculated for the treated groups at each assessment day and each time-point (1 h, 6 h and 12 h PI in study 1 and 2 h and 6 h PI in study 2, respectively), in accordance with the WAAVP guidelines, using the Abbott’s formula [12].

$$ \mathrm{Efficacy}\ \left(\%\right) = 100 \times \left({\mathrm{M}}_{\mathrm{c}}\hbox{--}\ {\mathrm{M}}_{\mathrm{t}}\right)/{\mathrm{M}}_{\mathrm{c}}, $$

where

M_c_ = arithmetic mean of live fleas on the negative control group (group 1)

M_t_ = arithmetic mean of live fleas on the Treatment-administration groups (groups 2 or 3)

#### Knock-down effect

The knock-down effect at 1 h PI (study 1) and 5 min PI (study 2) wt at 1 h PI (study 1) and 5up according to the formulas given below.

$$ \mathrm{Knock}\hbox{-} \mathrm{down}\ \left(\%\right) = \left[1\hbox{-} \left(100\ \hbox{--}\ \left(\mathrm{nt}\ \hbox{--}\ \mathrm{n}\mathrm{c}\right)\right)/100\ \right] \times 100, $$

where

nt = arithmetic mean number of moribund and dead fleas collected from trays underneath cages of each treated dog at 1 h PI (study 1) and 5 min PI (study 2).

nc = geometric or arithmetic mean number of moribund and dead fleas collected from trays underneath cages of each control dog at 1 h PI (study 1) and 5 min PI (study 2).

The statistical unit was the individual animal. The groups were compared at each time point in regard to speed of kill and knock-down effect by a one-way ANOVA with an administration effect on the untransformed live flea counts (speed of kill) or moribund/dead (knock-down effect) flea data. SAS Version 9.3 was used for all the statistical analyses.

## Results

### Study 1

#### Speed of kill

The arithmetic mean live flea counts recorded for the control group ranged from 47.2 to 85.5 over the one month assessment period indicating an adequate flea challenge at all assessment days and time points.

The efficacy results for study 1 are summarized in Table 1. The persistent speed of kill for the combination of fipronil and permethrin (Frontline Tri-Act®) (group 2) assessed 1 h after infestation from Days 7 to 28 ranged from 73.4 to 98.6 %. In comparison, the efficacy over the same assessment period for fipronil alone (Effipro®) (group 3) ranged from 0 to 43.4 %, markedly lower than that of the combination of fipronil and permethrin. Statistically significantly (*p* < 0.05) less fleas were observed in group 2 compared to group 3 on Days 7 to 28 at 1 h after infestation. There was no statistically significant (*p* > 0.05) difference in the number of fleas observed in group 2 compared to group 3 from Days 7 to 28 at 12 h after infestation (both products killed >99 % of fleas at 12 h).

**Table 1:** Study 1: Knock-down effect at 1 h and arithmetic mean flea counts and preventive efficacies at 1 h and 12 h (speed of kill) of the combination of permethrin and fipronil (group 2) and fipronil alone (group 3) based on arithmetic counts

	Knock-down effect (%) at 1 h		Mean flea counts and preventive efficacy (%)					
			at 1 h			at 12 h		
	Group 2	Group 3	Group 1	Group 2	Group 3	Group 1	Group 2	Group 3
	Fipronil/permethrin	Fipronil	Control	Fipronil/permethrin	Fipronil	Control	Fipronil/permethrin	Fipronil
D7	65.2 %^a^	7.8 %	85.5	1.2^a^ (98.6 %)	51.2 (40.2 %)	60.7	0.0 (100.0 %)	0.9 (99.0 %)
D14	67.7 %^a^	5.8 %	80.7	2.5^a^ (96.9 %)	45.7 (43.4 %)	61.5	0.0 (100.0 %)	0.0 (100.0 %)
D21	68.5 %^a^	4.2 %	76.5	5.3^a^ (93.0 %)	55.7 (27.2 %)	52.7	0.0 (100.0 %)	0.0 (100.0 %)
D28	58.2 %^a^	2.0 %	53.2	14.2^a^ (73.4 %)	62.0 (0.0 %)	47.2	0.3 (99.3 %)	0.2 (99.6 %)

^a^ statistically significant increased efficacy in the fipronil + permethrin group compared to the fipronil alone group (*p* < 0.05)

#### Knock-down effect

The arithmetic mean number of dead or moribund fleas collected from the control group ranged from 0.8 to 3.3 over the one month assessment period. The knock-down percentage for the combination of fipronil and permethrin (group 2) assessed 1 h after infestation from Days 7 to 28 ranged from 58.2 % to 68.5 %. In comparison, the knock-down percentage over the same assessment period for fipronil alone (group 3) ranged from 2.0 % to 7.8 %, markedly lower than that of the combination of fipronil and permethrin. Statistically significantly (*p* < 0.05) more fleas were knocked-down in group 2 compared to group 3 on Days 7 to 28 at 1 h after infestation.

### Study 2

#### Speed of kill

The arithmetic mean live flea counts recorded for the control group ranged from 81.8 to 97.3 over the one month assessment period indicating a vigorous flea challenge at all assessment days and time points.

The arithmetic mean efficacies (%) based on the 2 and 6 h flea count assessments are summarized in Table 2.

**Table 2:** Study 2: Knock-down effect at 5 min and arithmetic mean flea counts and preventive efficacies at 2 h and 6 h (speed of kill) of the combination of permethrin and fipronil (group 2) and a combination of dinetofuran + permethrin + pyriproxyfen (Group 3) compared to control (Group 1)

	Knock-down effect (%) at 5 min		Mean flea counts and preventive efficacy					
			at 2 h			at 6 h		
	Group 2	Group 3	Group 1	Group 2	Group 3	Group 1	Group 2	Group 3
	Fipronil + permethrin	Dinetofuran + permethrin + pyriproxyfen	Control	Fipronil + permethrin	Dinetofuran + permethrin + pyriproxyfen	Control	Fipronil + permethrin	Dinetofuran + permethrin + pyriproxyfen
D2	32.5 %^a^	17.1 %	94,8	9.6 (89.8 %)	26.3 (72.3 %)	89.8	0.6 (99.3 %)	0.8 (99.2 %)
D7	31.9 %^a^	6.6 %	88.6	0.9 (99.0 %)	8.1 (90.8 %)	87.8	0.0 (100.0 %)	0.9 (99.0 %)
D14	16.4 %^a^	3.8 %	84.5	3.3 (96.2 %)	9.5 (88.8 %)	81.8	0.3 (99.7 %)	1.8 (97.9 %)
D21	33.7 %^a^	10.1 %	97.3	5.4^a^ (94.5 %)	17.8 (81.7 %)	91.4	0.5 (99.5 %)	2.1 (97.7 %)
D28	29.9 %^a^	6.5 %	94.3	14.0 (85.1 %)	17.4 (81.6 %)	82.1	1.3 (98.5 %)	1.9 (97.7 %)

^a^ statistically significant increased efficacy in the fipronil/permethrin group compared to the dinetofuran + permethrin + pyriproxyfen group (*p* < 0.05)

At 2 h after infestation, the live flea counts recorded from the negative control group (group 1) were statistically significantly (*p* < 0.05) higher than for both group 2 and group 3 on all of the assessment days. At 2 h after infestation, the efficacy of the combination of fipronil and permethrin (85.1 to 99.0 %) was slightly higher than that of the combination of permethrin, dinetofuran and pyriproxyfen (72.3 to 90.8 %) for the duration of the study. Statistically significant differences (*p* = 0.0166) were recorded between the two treated groups (group 2 and group 3) at 2 h after infestation on Day 21, where significantly less fleas were recorded for group 2 compared to group 3.

At 6 h after infestation, the live flea counts recorded from the negative control group were statistically significantly (*p* < 0.05) higher than those of both group 2 and group 3 on all of the assessment days. At 6 h after infestation, the efficacy of the combination of fipronil and permethrin (98.5 to 100 %) was comparable to that of the combination of permethrin, dinetofuran and pyriproxyfen (97.7 to 99.2 %) for the duration of the study. No statistically significant (*p* < 0.05) differences were recorded between the two treated groups at 6 h after infestation.

#### Knock-down effect

The arithmetic mean number of dead and moribund fleas collected from trays underneath cages at 5 min and the knock-down (%) based on these numbers are summarised in Table 2.

No dead or moribund fleas were collected from the control group over the study period. The dead and moribund flea counts differed statistically significantly (*p* < 0.05) for group 2 (combination of fipronil and permethrin) and group 3 (combination of permethrin, dinetofuran and pyriproxyfen) compared to the control group on all of the assessment days.

Statistically significantly (*p* < 0.05) more fleas were knocked-down in group 2 compared to group 3 on all of the assessment days. The knock-down (%) against flea infestations 5 min after infestation ranged from 16,4 to 33.7 % from Days 2 to 28 for the combination of fipronil and permethrin (group 2) and from 3.8 to 17.1 % for the combination of permethrin, dinetofuran and pyriproxyfen (group 3).

## Discussion

These two studies demonstrate the high level of rapid preventive efficacy of the spot on combination of fipronil and permethrin in Frontect®/Frontline Tri-Act® formulation against flea infestations. For a whole month, a significant knock-down of infesting *C. felis* fleas is obtained as soon as 5 min after infestation. The complete efficacy (>95 % as defined by guidelines [11, 12] is reached in 1 h (study 1) or 2 h (study 2) PI for 14 days and in 6 h PI for the whole month. Efficacy remains >85 % at 2 h PI for the whole months.

In addition, the comparison of the preventive efficacy against flea infestation of the combination of fipronil and permethrin with those of either fipronil alone or a synergistic combination of permethrin, dinetofuran, and pyriproxyfen demonstrated a significantly higher efficacy of the fipronil and permethrin combination at the earliest time counts for the whole duration of the study (1 h knock-down effect and acaricidal efficacy against fipronil alone and 5 min knock-down effect against the combination of permethrin + dinetofuran + pyriproxyfen). At later time points, the level of efficacy is high for all products and therefore differences are not significant anymore even if the permethrin + fipronil combination offers slightly better efficacy results.

The present studies focused on preventive efficacy. Indeed, due to the time requested for the diffusion of the product over the entire body of the animal, maximal efficacy and speed of kill are reached after 24 h post treatment.

Previous studies have demonstrated that 10 to 25 % of fleas start to feed within 5 min PI [7–9]. The preventive speed of kill is therefore a major element for flea control. In addition, this level of preventive efficacy could likely affect the transmission of pathogens through blood feeding and through the inhibition of the excretion of potentially infectious faeces by the adult fleas. This rapid effect is also expected to reduce the severity of allergic dermatitis associated with flea bites. The rapid knock-down effect and speed of kill of the formulation demonstrated over the whole month period appear therefore to be key benefits for the control of flea burden in dogs.

A similar enhanced speed of kill has been obtained against tick infestations. The combination product offers a complete preventive protection against *Ixodes ricinus* and *Rhipicephalus sanguineus* ticks in 6 h for a whole month [17]. This level of tick efficacy reduces the risk for transmission of both *Babesia canis* by *Dermacentor reticulatus* ticks and *Ehrlichia canis* by *R. sanguineus* [18].

## Conclusion

The sustained rapidity of action of the combination of permethrin and fipronil in frontline tri-act®/frontect® formulation over the whole month period may suggest synergy between the actives. This hypothesis can be investigated through in vitro studies for a better characterization. The rapid flea knock-down effect and speed of kill of the formulation provide a reliable strategy against flea infestations in dogs. Further studies should focus on the ability of the formulation to reduce the risk for flea-borne diseases transmission.
